# Microbiologically Influenced Corrosion of 2707 Hyper-Duplex Stainless Steel by Marine *Pseudomonas aeruginosa* Biofilm

**DOI:** 10.1038/srep20190

**Published:** 2016-02-05

**Authors:** Huabing Li, Enze Zhou, Dawei Zhang, Dake Xu, Jin Xia, Chunguang Yang, Hao Feng, Zhouhua Jiang, Xiaogang Li, Tingyue Gu, Ke Yang

**Affiliations:** 1 School of Metallurgy, Northeastern University, Shenyang, 110819, China; 2 Corrosion and Protection Center, University of Science and Technology Beijing, Beijing 100083, P. R. China; 3 Institute of Metal Research, Chinese Academy of Sciences, Shenyang, 110016, China; 4 College of Chemistry, Liaoning University, Shenyang, 110036, China; 5 Department of Chemical and Biomolecular Engineering, Institute for Corrosion and Multiphase Technology, Ohio University, Athens, Ohio 45701, USA

## Abstract

Microbiologically Influenced Corrosion (MIC) is a serious problem in many industries because it causes huge economic losses. Due to its excellent resistance to chemical corrosion, 2707 hyper duplex stainless steel (2707 HDSS) has been used in the marine environment. However, its resistance to MIC was not experimentally proven. In this study, the MIC behavior of 2707 HDSS caused by the marine aerobe *Pseudomonas aeruginosa* was investigated. Electrochemical analyses demonstrated a positive shift in the corrosion potential and an increase in the corrosion current density in the presence of the *P. aeruginosa* biofilm in the 2216E medium. X-ray photoelectron spectroscopy (XPS) analysis results showed a decrease in Cr content on the coupon surface beneath the biofilm. The pit imaging analysis showed that the *P. aeruginosa* biofilm caused a largest pit depth of 0.69 μm in 14 days of incubation. Although this was quite small, it indicated that 2707 HDSS was not completely immune to MIC by the *P. aeruginosa* biofilm.

Duplex stainless steel (DSS) is widely used in various industries due to its desirable combination of excellent mechanical properties and corrosion resistance^1^,^2^. However, localized pitting corrosion can still occur and it affects the integrity of this kind of steel^3^,^4^. DSS is not immune to microbiologically influenced corrosion (MIC)^5^,^6^. Despite a very broad range of applications for DSS, there are still environments where the corrosion resistance of DSS is inadequate for long-term services. This means, more expensive materials with higher corrosion resistance are needed. Jeon *et al.*^7^ found that even super duplex stainless steels (SDSSs) exhibit some limits in corrosion resistance. Therefore, hyper duplex stainless steels (HDSSs) with a higher corrosion resistance are needed in some applications. This led to the development of highly alloyed HDSSs.

The corrosion resistance of DSS is determined by the ratio of the α-phase and γ-phase and by the Cr, Mo, and W depleted regions that are adjacent to the secondary phases^8^,^9^,^10^. HDSS contains high levels of Cr, Mo and N^11^, resulting in its excellent corrosion resistance and a high value (45–50) of pitting resistance equivalent number (PREN), which is calculated from wt.% Cr + 3.3 (wt.% Mo + 0.5 wt.% W) + 16 wt.% N^12^. Its excellent corrosion resistance properties rely on a well-balanced composition with approximately 50% ferrite (α) and 50% austenite (γ) phases, that offer HDSS improved mechanical properties and higher chloride corrosion resistance compared with conventional DSS^13^. The improved corrosion resistance extends the use of HDSS in more aggressive chloride environments, such as marine environments.

MIC is a major problem in many industries such as oil and gas, as well as water utilities^14^. MIC accounts for 20% of all corrosion damages^15^. MIC is bioelectrochemical corrosion that can be observed in many environments^16^. Biofilms formed on a metal surface will change the electrochemical conditions and thus influencing the corrosion processes. It is widely accepted that MIC corrosion is caused by biofilms^14^. Electrogenic microbes corrode metals in order to obtain maintenance energy for survival^17^. The most recent MIC research suggested that EET (extracellular electron transfer) is a rate-limiting factor in MIC caused by electrogenic microbes. Zhang *et al.*^18^ demonstrated that an electron mediator accelerated the electron transfer between sessile *Desulfovibrio vulgaris* cells and 304 stainless steel, resulting in a much more severe MIC attack. Enning *et al.*^19^ and Venzlaff *et al.*^20^ showed that a corrosive sulfate-reducing bacterium (SRB) biofilm was capable of directly uptaking electrons from the metal matrix, resulting in serious pitting corrosion.

DSS is known to be susceptible to MIC in environments containing SRB, iron reducing bacteria (IRB), etc^21^. These bacteria cause localized pitting corrosion on DSS surfaces under biofilms^22^,^23^. Unlike DSS, very little is known for the MIC of HDSS^24^.

*Pseudomonas aeruginosa* is a Gram-negative motile rod bacterium widely distributed in nature^25^. *P. aeruginosa* is also a predominant group of micro-organisms in marine environments causing MIC to steels^26^. The genus *Pseudomonas* is closely involved in corrosion processes and has been recognized as the pioneer colonizer in the process of biofilm formation^27^. Mahat *et al.*^28^ and Yuan *et al.*^29^ demonstrated that *P. aeruginosa* has the propensity to increase the corrosion rates of mild steels and alloys in aquatic environments.

The main aim of this work was to investigate the MIC characteristics of 2707 HDSS caused by the marine aerobe *P. aeruginosa* using electrochemical methods, surface analysis techniques and corrosion product analyses. Electrochemical studies, including open circuit potential (OCP), linear polarization resistance (LPR), electrochemical impedance spectroscopy (EIS) and potential dynamic polarization were performed to study the MIC behavior of 2707 HDSS. The energy dispersive spectrometer (EDS) analysis was undertaken to find the chemical elements on the corroded surface. In addition, the X-ray photoelectron spectroscopy (XPS) analysis was used to determine the stability of the oxide film passivity under the influence of a marine environment containing *P. aeruginosa.* The pit depth was measured under a confocal laser scanning microscope (CLSM).

## Results

### Material characterizations

**Figure 1:**
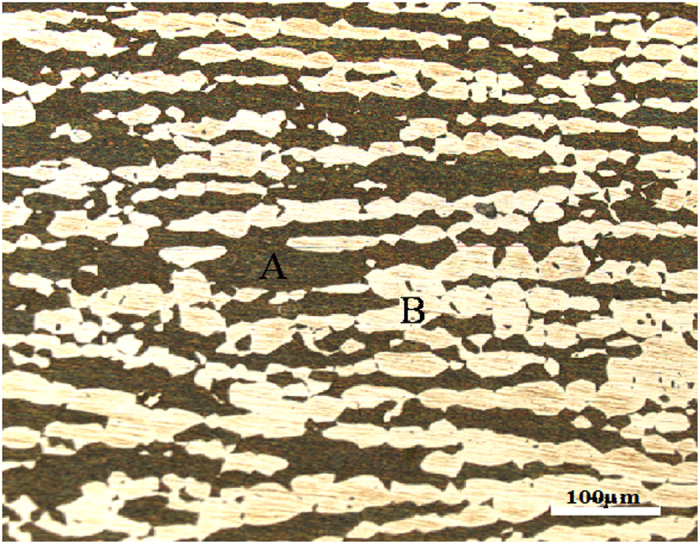
Microstructure of 2707 HDSS: (**A**) ferrite, and (**B**) austenite.

Table 1 lists the chemical composition of 2707 HDSS. Table 2 shows that 2707 HDSS has excellent mechanical properties with yield strength of 650 MPa. Figure 1 reveals the optical microstructures of the solution heat-treated 2707 HDSS. Elongated bands of austenite and ferrite phases without secondary phases can be seen in the microstructure that contains approximately 50% austenite and 50% ferrite phases.

### Open circuit potential versus time

**Figure 2:**
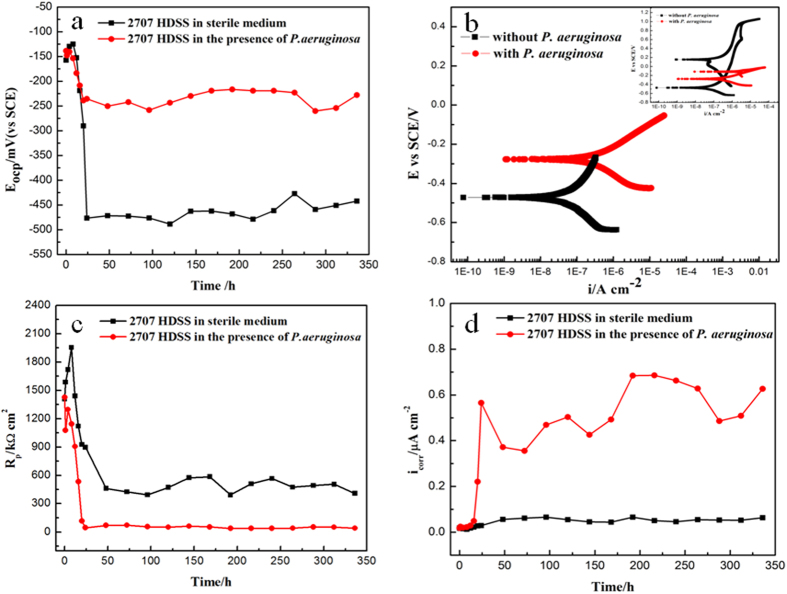
Electrochemical tests for 2707 HDSS coupons in the abiotic medium and in the *P. aeruginosa* broth at 37 °C: (**a**) variation of E_ocp_ with exposure time, (**b**) polarization curves on the 14^th^ day, (**c**) variation of R_p_ with exposure time, and (**d**) variation of i_corr_ with exposure time.

Figure 2a shows open circuit potential (E_ocp_) vs. exposure time data for 2707 HDSS in the abiotic 2216E medium and in the *P. aeruginosa* broth at 37 °C for 14 days. It reveals that the most and significant variation in E_ocp_ occurred in the initial 24 h. The E_ocp_ values in both cases peaked around −145 mV (vs. SCE) at about 16 h and then dropped sharply, reaching −477 mV (vs. SCE) and −236 mV (vs. SCE) for the abiotic coupon and the *P. aeruginosa* coupon, respectively. After 24 h, the E_ocp_ value of 2707 HDSS remained relatively stable at −228 mV (vs. SCE) for the *P. aeruginosa* coupon, while the corresponding value for the abiotic coupon was approximately −442 mV (vs. SCE). The E_ocp_ in the presence of *P. aeruginosa* was considerably lower.

### Polarization Curves

The values of the electrochemical corrosion parameters of 2707 HDSS coupons after exposure for 14 days in the abiotic medium as well as in the *P. aeruginosa* inoculated medium are listed in Table 3. The tangential lines of the anodic and cathodic curves were extrapolated to reach an intersection point to yield corrosion current density (i_corr_), corrosion potential (E_corr_), and Tafel slopes (β_α_ and β_c_) following the standard approach^30^,^31^.

As shown in Figure 2b, compared with the abiotic curves, the *P. aeruginosa* curves shifted upward leading to an increase of E_corr_. The i_corr_ value, that was directly proportional to the corrosion rate, increased to 0.328 μA cm^−2^ for the *P. aeruginosa* coupon, four times greater than that for the abiotic coupon (0.087 μA cm^−2^).

### Linear polarization resistance

LPR is a classical electrochemical method for fast corrosion analysis that is non-destructive. It is also used to study MIC^32^. Figure 2c shows the variation of the polarization resistance (R_p_) as a function of exposure time. A higher R_p_ value means less corrosion. During the initial 24 h, the R_p_ of 2707 HDSS reached a maximum value of 1955 kΩ cm^2^ for the abiotic coupon and 1429 kΩ cm^2^ for the *P. aeruginosa* coupon. Figure 2c also shows that the R_p_ value fell quickly after one day and then remained relatively unchanged for the next 13 days. The R_p_ value for the *P. aeruginosa* coupon was around 40 kΩ cm^2^, much lower than the 450 kΩ cm^2^ value for the abiotic coupon.

The i_corr_ value is directly proportional to the uniform corrosion rate. Its value can be calculated from the Stern-Geary equation below,


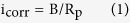


Following Zou *et al.*^33^, the Tafel slope B in this work was assumed a typical value of 26 mV/dec. Figure 2d shows that i_corr_ remained relatively stable for the abiotic 2707 coupon while it fluctuated considerably for the *P. aeruginosa* coupon that had a large jump after the initial 24 h. The i_corr_ value for the *P. aeruginosa* coupon was one order of magnitude higher than that of the abiotic control. This trend agreed with the polarization resistance result.

### Electrochemical impedance spectroscopy

EIS is another nondestructive technique to characterize electrochemical reactions at a corrosion interface^34^. Impedance spectra and calculated capacitance values of coupons exposed to the abiotic medium and the *P. aeruginosa* solution, R_b_ the resistance of passive film/biofilm formed on the coupon surface, R_ct_ the charge transfer resistance, C_dl_ the capacitance of the electrical double layer (EDL), and Q_CPE_ the constant phase element (CPE) parameter. These parameters were further analyzed by fitting data with an equivalent electrical circuit (EEC) model.

**Figure 3:**
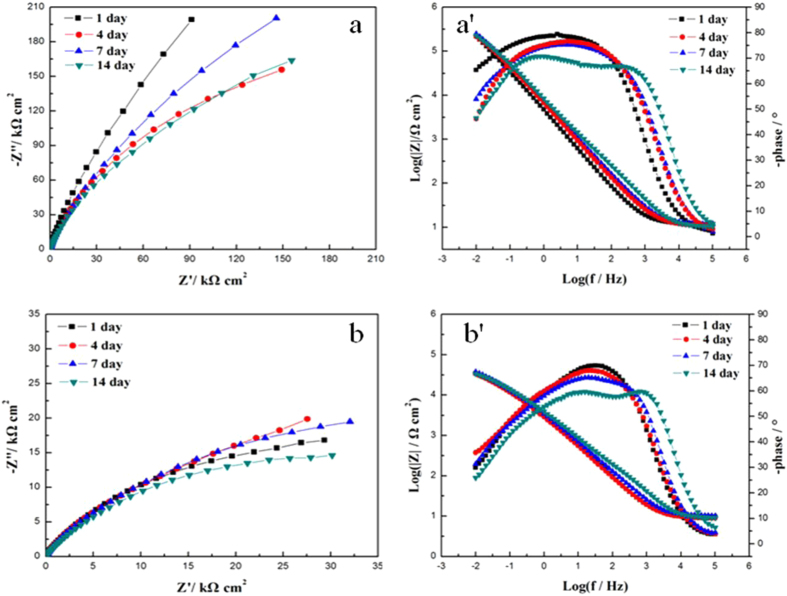
The Nyquist and Bode plots of 2707 HDSS coupons with and without exposure to *P. aeruginosa*: (**a**,**a′**) 2707 HDSS in the abiotic medium, and (**b**,**b′**) 2707 HDSS in the *P. aeruginosa* broth.

**Figure 4:**
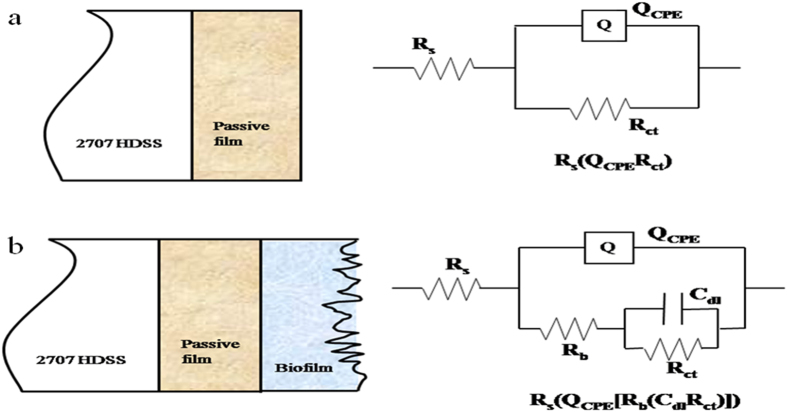
Two physical models and the corresponding equivalent circuits used for fitting the impedance spectra of 2707 HDSS coupons: (**a**) in the abiotic medium, and (**b**) in the *P. aeruginosa* broth.

Figure 3 shows the typical Nyquist diagrams (a and b) and Bode plots (a′ and b′) at different incubation times for 2707 HDSS coupons in the abiotic medium and in the *P. aeruginosa* broth. The diameters of the Nyquist loops decreased in the presence of *P. aeruginosa*. The Bode plot (Figure 3b′) shows an increase of the total impedance magnitudes. The information of the relaxation time constants can be provided by the phase maxima. Figure 4 shows the physical structure and their corresponding EECs based on a single-layer (a) and a double-layer (b). CPE was introduced into the EEC models. Its admittance and impedance, respectively, are expressed below:


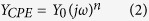



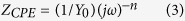


where Y_0_ is the magnitude of the CPE, j the imaginary number or (−1)^1^/2, ω the angular frequency, and n the CPE power index which is less than unity^35^. The inverse of the charge-transfer resistance (i.e., 1/R_ct_) corresponds to the corrosion rate. A smaller R_ct_ means a faster corrosion rate^27^. After 14 days of incubation, the R_ct_ of the *P. aeruginosa* coupon reached 32 kΩ cm^2^, much smaller than the 489 kΩ cm^2^ for the abiotic coupon (Table 4).

### Biofilm visualization

**Figure 5:**
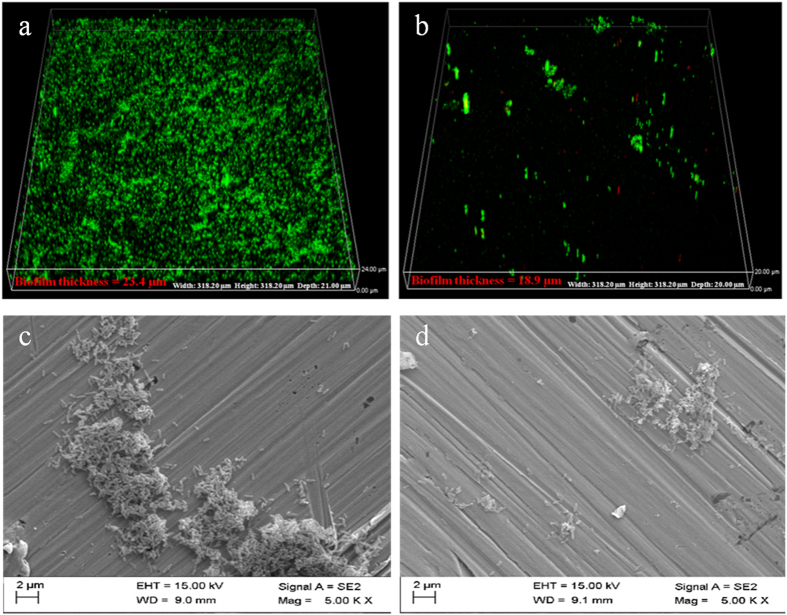
The morphology of *P. aeruginosa* biofilms on coupon surfaces: (**a**) the 3-D CLSM image after 7 days, (**b**) the 3-D CLSM image after 14 days, (**c**) the SEM image after 7 days, and (**d**) the SEM image after 14 days.

The CLSM images and SEM images in Figure 5 clearly indicate that the biofilm coverage on the 2707 HDSS coupon surface after 7 days was dense. However, after 14 days the biofilm coverage was sparse and some dead cells appeared. Table 5 shows the biofilm thickness on the 2707 HDSS coupon after exposure to *P. aeruginosa* for 7 and 14 days. The largest biofilm thickness changed from 23.4 μm after 7 days to 18.9 μm after 14 days. The average biofilm thickness also confirmed the trend. It decreased from 22.2 ± 0.7 μm after 7 days to 17.8 ± 1.0 μm after 14 days.

**Figure 6:**
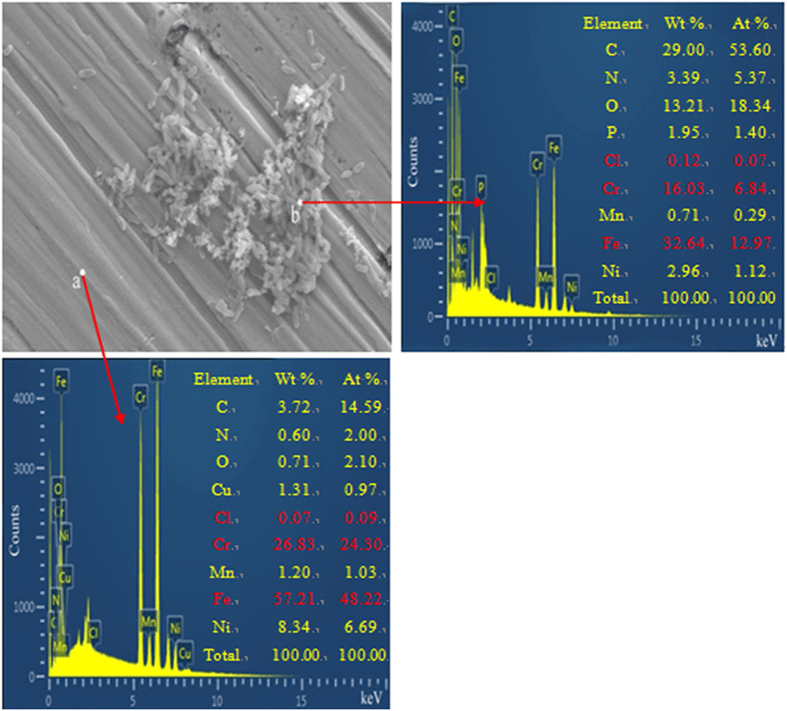
Results of EDS analysis of the 2707 HDSS surface after exposure to *P. aeruginosa* for 14 days.

Chemical elements in the biofilm and corrosion products on the coupon exposed to *P. aeruginosa* for 14 days were revealed by EDS. Figure 6 shows the amounts of C, N, O, P in the biofilm and corrosion products were much higher than those in the bare metal because these elements were associated with the biofilm and its metabolic products. Cr and Fe are needed by microorganisms only at trace levels. The high levels of Cr and Fe in the biofilm and corrosion products on the coupon surface suggest loss of the elements by the metal matrix due to corrosion.

**Figure 7:**
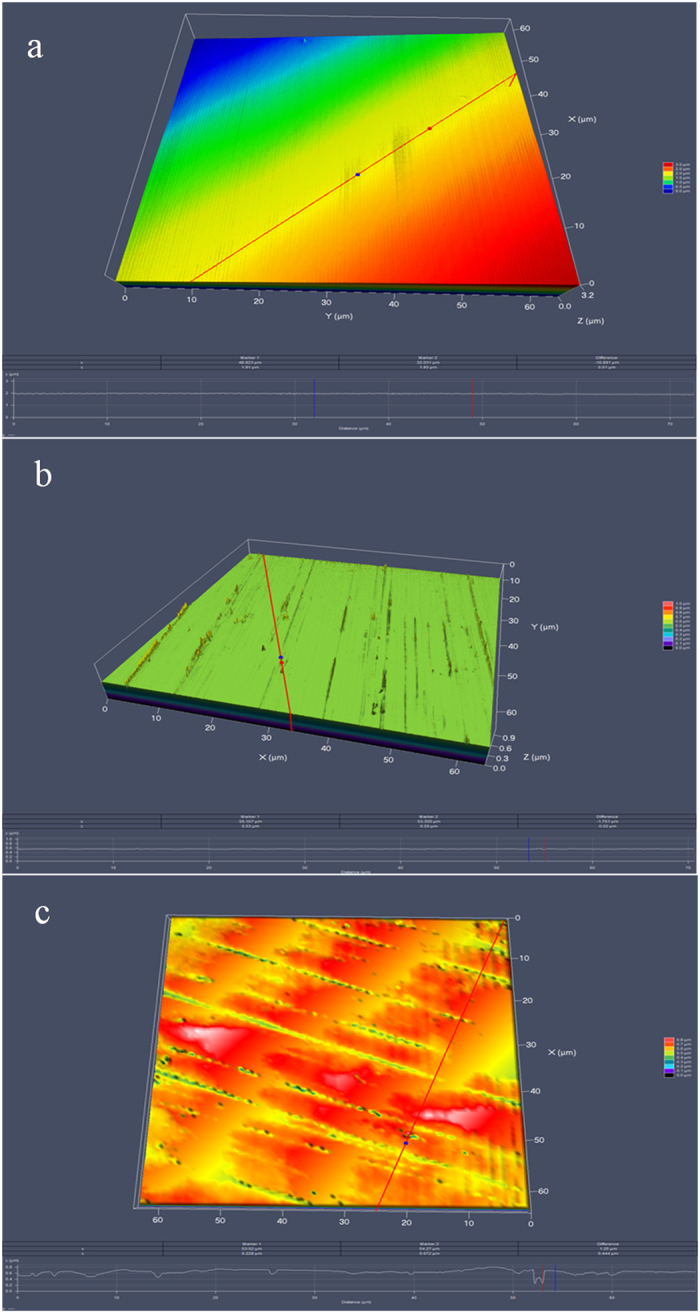
CLSM analysis of pits on coupon surfaces: (**a**) before exposure, (**b**) in the abiotic medium for 14 days, and (**c**) in the *P. aeruginosa* broth for 14 days.

Pitting corrosion was observed with and without *P. aeruginosa* in the 2216E medium after 14 days. Before incubation, the coupon surfaces were smooth and had no defects (Figure 7a). After incubation and removing the biofilm and corrosion products, the deepest pits on the coupon surfaces were examined under CLSM as shown in Figure 7b and c. No significant pits were found on the abiotic control coupon surface (largest pit depth 0.02 μm). The largest pit depth caused by *P. aeruginosa* was 0.52 μm after 7 days and 0.69 μm after 14 days, and the average largest pit depth based on 3 coupons (with 10 largest pit depth values per coupon selected) reached 0.42 ± 0.12 μm and 0.52 ± 0.15 μm, respectively (Table 5). These pit depth values were small, but significant.

### XPS analysis of 2707 HDSS coupon surfaces

**Figure 8:**
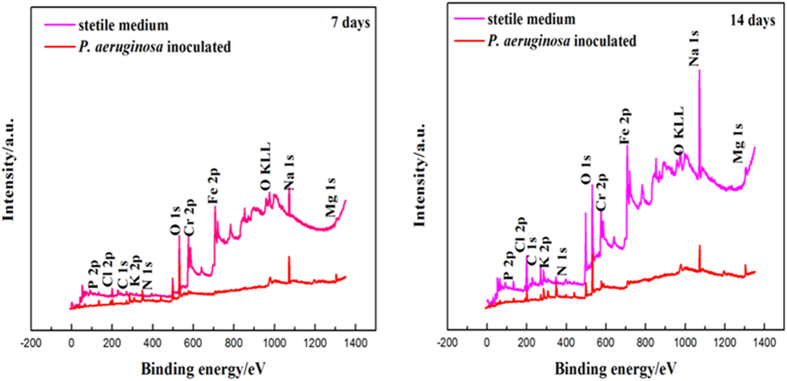
The wide XPS spectra for the surfaces of 2707 HDSS coupons in the both media for 7 days and 14 days, respectively.

**Figure 9:**
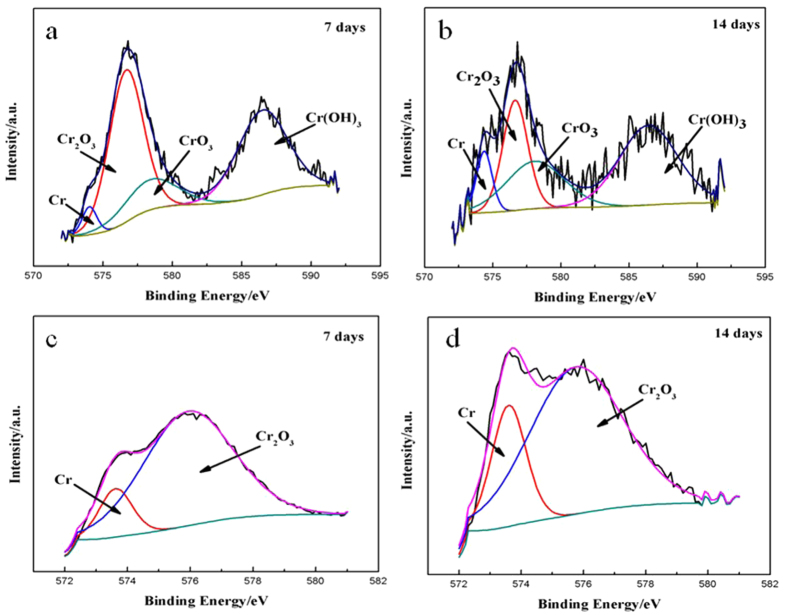
The high resolution XPS spectra of Cr 2p for 2707 HDSS: (**a**) with exposure to *P. aeruginosa* for 7 days, (**b**) with exposure to *P. aeruginosa* for 14 days, (**c**) in the abiotic medium for 7 days, and (**d**) in the abiotic medium for 14 days.

Figure 8 shows the XPS spectra of different coupon surfaces, and the chemical composition of each surface analysis is summarized in Table 6. In Table 6, the atomic percentages of Fe and Cr in the presence of *P. aeruginosa* (samples A and B) were much lower than those for the abiotic control coupons (samples C and D). For the *P. aeruginosa* coupons, the Cr 2p core-level spectrum was curve-fitted into four peak components at binding energy (BE) values of 574.4, 576.6, 578.3 and 586.8 eV, which were attributable to Cr, Cr_2_O_3_, CrO_3_ and Cr(OH)_3_, respectively (Figure 9a and b). For the abiotic coupons, the Cr 2p core-level spectrum contained two dominant peaks for Cr (BE at 573.80 eV) and Cr_2_O_3_ (BE at 575.90 eV) in Figure 9c and d, respectively. The most noticeable difference between the abiotic coupons and the *P. aeruginosa* coupons was the presence of Cr^6+^ beneath the biofilm and a higher relative portion of Cr(OH)_3_ (BE at 586.8 eV).

## Discussion

HDSSs exhibit a high level of corrosion resistance in most environments. Kim *et al.*^2^ reported that UNS S32707 HDSS is defined as a highly alloyed DSS with a PREN in excess of 45. The 2707 HDSS coupons in this work had a PREN value of 49. This is due to its high Cr content and elevated Mo and Ni levels, which are beneficial in acid and high chloride environments. Furthermore, the well-balanced composition and defect-free microstructure are useful for structural stability and corrosion resistance. Despite its superior chemical corrosion resistance, however, the experimental data in this work demonstrated that 2707 HDSS was not completely immune to the MIC by the *P. aeruginosa* biofilm.

The electrochemical results revealed that the corrosion rate of 2707 HDSS significantly increased in the *P. aeruginosa* broth after 14 days compared to the abiotic medium. In Figure 2a, a decrease of E_ocp_ was observed in both the abiotic medium and the *P. aeruginosa* broth in the initial 24 h. Afterwards, the biofilm completed the coverage of the coupon surface, E_ocp_ became relatively stable^36^. However, the biotic E_ocp_ was at a much higher level than the abiotic E_ocp_. It is reasonable to believe that the difference was due to the formation of the *P. aeruginosa* biofilm. In Figure 2d, the i_corr_ value of 2707 HDSS reached 0.627 μA cm^−2^ in the presence of *P. aeruginosa*, one order of magnitude higher than that for the abiotic control (0.063 μA cm^−2^), which was consistent with the R_ct_ values measured by EIS. In the first few days, the impedance value increased in the *P. aeruginosa* broth due to the attachment of *P. aeruginosa* cells and the formation of the biofilm. However, the impedance then decreased when the biofilm fully covered the coupon surface. The protective layer was first attacked because of the formation of the biofilm and metabolites of the biofilm. Consequently, the corrosion resistance decreased with time and the *P. aeruginosa* attachment caused the localized corrosion. The tendency in the abiotic medium was different. The corrosion impedance of the abiotic control was much higher than the corresponding value of the coupon exposed to the *P. aeruginosa* broth. Furthermore, for the abiotic samples, the R_ct_ value of 2707 HDSS reached 489 kΩ cm^2^ on the 14^th^ day, fifteen times larger than that in the presence of *P. aeruginosa* (32 kΩ cm^2^). Thus, 2707 HDSS possessed excellent corrosion resistance in the sterile environment, but it was not immune to the MIC attack by the *P. aeruginosa* biofilm.

These results were also observed from the polarization curves in Figure 2b. The anodic branch was attributed to the formation of *P. aeruginosa* biofilm and the metal oxidation reaction. Meanwhile the cathodic reaction was the reduction of oxygen. The presence of *P. aeruginosa* greatly increased the corrosion current density, which was approximately one order of magnitude higher than the abiotic control. It suggested that the *P. aeruginosa* biofilm increased the localized corrosion of 2707 HDSS. Yuan *et al.*^29^ found that the 70/30 Cu-Ni alloy corrosion current density increased under the attack of a *P. aeruginosa* biofilm. It could be due to the *P. aeruginosa* biofilm’s biocatalysis of the reduction of oxygen^37^. This observation could explain the MIC of 2707 HDSS in this work as well. It was also possible that the aerobic biofilm made oxygen less available underneath it. Thus, the failure to repassivate the metal surface by oxygen could be a contributing factor in the MIC in this work.

Dickinson *et al.*^38^ proposed that the rates of chemical and electrochemical reactions can be directly affected by the metabolic activities of the sessile bacteria on a coupon surface and the nature of corrosion products. As seen in Figure 5 and Table 5, both the cell number and biofilm thickness decreased after 14 days. This can be reasonably explained that after 14 days, most of the sessile cells on the 2707 HDSS surface were dead due to the exhaustion of the nutrients in the 2216E medium or the toxic metal ions released from the 2707 HDSS matrix. This was a limitation of batch experiments.

In this work, the *P. aeruginosa* biofilm promoted the localized depletion of Cr and Fe underneath the biofilm on the 2707 HDSS surface (Figure 6). In Table 6, compared with sample C, there was a decrease of Fe and Cr in sample D, indicating that dissolution Fe and Cr caused by the *P. aeruginosa* biofilm continued beyond the first 7 days. The 2216E medium was used to simulate the marine environment. It contained 17700 ppm Cl^−^, comparable to that in the natural seawater. The presence of 17700 ppm Cl^−^ was the main reason responsible for the decrease of Cr from the 7-day and 14-day abiotic samples analyzed by XPS. Compared with the *P. aeruginosa* coupons, the dissolution of Cr in abiotic coupons was much smaller due to the strong Cl^−^ resistance of 2707 HDSS in an abiotic environment. Figure 9 shows that the presence of Cr^6+^ in the passivation film. It might be involved in the removal of Cr from the steel surface by the *P. aeruginosa* biofilm as suggested by Chen and Clayton^39^.

The pH values of the culture medium before and after incubation were 7.4 and 8.2, respectively, due to bacterial growth. Thus, underneath the *P. aeruginosa* biofilm, organic acid corrosion was unlikely a contributing factor in this work due to the relatively high pH values in the bulk medium. The pH value of the abiotic control medium didn’t change significantly (from the initial 7.4 to the final 7.5) in the 14-day test duration. The increase of pH in the inoculated medium after incubation was due to the metabolic activities of *P. aeruginosa*, which was found to have the same impact on pH in the absence of coupons.

As shown in Figure 7, the maximum pit depth caused by the *P. aeruginosa* biofilms was 0.69 μm, which was much larger than that in the abiotic medium (0.02 μm). This was consistent with the electrochemical data above. The 0.69 μm pit depth was more than ten times smaller than the 9.5 μm value reported for 2205 DSS under the same conditions^40^. These data proved that 2707 HDSS exhibited a better MIC resistance compared with 2205 DSS. This should not be a surprise because 2707 HDSS has a higher Cr level that offers more durable passivity, making it more difficult for *P. aeruginosa* to depassivate and initiate pitting corrosion due to the balanced phase structure without harmful secondary precipitates^41^.

In conclusion, MIC pitting corrosion was found on the surface of 2707 HDSS in the *P. aeruginosa* broth compared to negligible pitting in the abiotic medium. This work demonstrated that 2707 HDSS had much better MIC resistance than 2205 DSS, but was not completely immune to MIC due to the *P. aeruginosa* biofilm. These findings are useful in the selection of a suitable stainless steel for the marine environment and in the estimation of service life span.

## Methods

### Coupon preparation

The coupons of 2707 HDSS were provided by School of Metallurgy, Northeastern University (NEU) in Shenyang, China. The elemental composition of 2707 HDSS was shown in Table 1, which was analyzed by the Department of Materials Analysis and Testing of NEU. All specimens were solution-treated at 1180 °C for 1 h. Prior to the corrosion tests, coin-shaped 2707 HDSS with a top exposed surface area of 1 cm^2^ were polished to 2000 grit with silicon carbide papers, and then further polished with a 0.05 μm Al_2_O_3_ powder suspension. The side and bottom surface were protected by an inert paint. After drying, the coupons were rinsed with sterile deionized water, and sterilized with 75% (v/v) ethanol for 0.5 h. They were then air dried under ultraviolet (UV) light for 0.5 h before use.

### Bacterial strain and cultivation

The marine *P. aeruginosa* MCCC 1A00099 strain was purchased from the Marine Culture Collection of China (MCCC), Xiamen, China. The marine 2216E liquid medium (Qingdao Hope Bio-technology Co., Qingdao, China) was used to culture the *P. aeruginosa* at 37 °C aerobically in 250 ml flasks and in a 500 ml electrochemical glass cell. The culture medium contained (g/L): 19.45 NaCl, 5.98 MgCl_2_, 3.24 Na_2_SO_4_, 1.8 CaCl_2_, 0.55 KCl, 0.16 Na_2_CO_3_, 0.08 KBr, 0.034 SrCl_2_, 0.08 SrBr_2_, 0.022 H_3_BO_3_, 0.004 NaSiO_3_, 0.0024 NaF, 0.0016 NH_4_NO_3_, 0.008 NaH_2_PO_4_, 5.0 peptone, 1.0 yeast extract, and 0.1 ferric citrate. It was autoclave-sterilized at 121 °C for 20 min prior to inoculation. A hemocytometer under a light microscope at 400× magnification was used to enumerate sessile and planktonic cells. The initial planktonic *P. aeruginosa* cell concentration immediately after inoculation was approximately 10^6^ cells/ml.

### Electrochemical test procedures

Electrochemical tests were carried out in a classical three-electrode glass cell with a culture medium volume of 500 ml. A platinum sheet and a saturated calomel electrode (SCE) connected to the reactor via a Luggin capillary filled with a salt bridge as the counter and reference electrodes, respectively. To create a working electrode, a rubber-coated copper wire was connected to each coupon and covered with epoxy resin leaving an exposed one-sided surface area of approximately 1 cm^2^ for this working electrode. During electrochemical measurements, samples were placed in the 2216E medium, and maintained at a constant incubation temperature (37 °C) in a water bath. The OCP, LPR, EIS and potential dynamic polarization data were measured with an Autolab potentiostat (Reference 600^TM^, Gamry Instruments, Inc., USA). LPR tests were recorded at a scan rate of 0.125 mV s^−1^ in the range of −5 and 5 mV versus E_ocp_, and the sampling frequency was 1 Hz. EIS was performed under a steady-state E_ocp_ using a 5 mV applied voltage at sinusoidal wave in a frequency range of 0.01 to 10,000 Hz. Before the potential scan, the electrodes were in the open circuit mode until a steady free corrosion potential value reached^42^. The polarization curves were then run at a scan rate of 0.166 mV/s from −0.2 to 1.5 V vs. E_ocp_. Each test was repeated three times with and without *P. aeruginosa*.

### Surface analysis

The coupons for the metallographic analysis were mechanically polished with wet SiC paper of 2000 grit, and then further polished with a 0.05 μm Al_2_O_3_ powder suspension to enable optical observations to be made. Metallographic analysis was carried out using an optical microscopy. The coupons were etched with a 10 wt.% potassium hydroxide solution^43^.

After incubation, a coupon was washed with a phosphate buffer saline (PBS) solution (pH 7.4 ± 0.2) three times and then fixed with 2.5% (v/v) glutaraldehyde for 10 h to fix the biofilm. It was subsequently dehydrated with a graded series (50%, 60%, 70%, 80%, 90%, 95% and 100% v/v) of ethanol before air-drying. Finally, the coupon surface was sputter-coated with a gold film to provide conductivity for SEM observation^44^. SEM images focused on spots with the most sessile *P. aeruginosa* cells on each coupon surface. The EDS analysis was undertaken to find the chemical elements. A Zeiss confocal laser scanning microscope (CLSM) (LSM 710, Zeiss, Germany) was used to measure the pit depth. To observe the corrosion pits underneath a biofilm, the coupon was first cleaned according to the Chinese National Standards (CNS) GB/T4334.4-2000 to remove the corrosion products and biofilms on the coupon surface.

An X-ray photoelectron spectroscopy (XPS, ESCALAB250 surface analysis system, Thermo VG, USA) analysis was performed using a monochromatic X-ray source (an aluminum Kα line of 1500 eV energy and 150 W power) within the wide binding energy range of 0–1350 eV under standard conditions. The high resolution spectra were recorded using 50 eV pass energy and 0.2 eV step.

### Bacterial stain assay

The coupons after incubation were taken out and gently rinsed with PBS (pH 7.4 ± 0.2) for 15 s^45^. In order to observe the bacterial viability of the biofilms on the coupon, LIVE/DEAD *Bac*Light Bacterial Viability Kits (Invitrogen, Eugene, OR, USA) were used to stain the biofilms. The kits have two fluorescent dyes, the green-fluorescent SYTO-9 dye and the red-fluorescent propidium iodide (PI) dye. Under CLSM, the dots with fluorescent green and red represented the live and dead cells, respectively. When staining, 1 ml mixture containing 3 μl SYTO-9 and 3 μl PI solutions were incubated for 20 min in the dark at room temperature (23 ^o^C). After that, a Nikon CLSM machine (C2 Plus, Nikon, Japan) was used to observe the stained sample at at two wave lengths (488 nm for living cells and 559 nm for dead cells). The biofilm thickness was measured in the 3-D scanning mode.

## Additional Information

**How to cite this article**: Li, H. *et al.* Microbiologically Influenced Corrosion of 2707 Hyper-Duplex Stainless Steel by Marine *Pseudomonas aeruginosa* Biofilm. *Sci. Rep.* **6**, 20190; doi: 10.1038/srep20190 (2016).

## Figures and Tables

**Table 1:** Chemical composition of 2707 HDSS.

Material	Element										
	Si	Mn	P	S	Co	Cr	Ni	Mo	Cu	N	Fe
2707 HDSS (wt.%)	0.42	1.11	0.005	0.003	1.00	26.83	7.14	4.88	0.98	0.39	Bal.

**Table 2:** Mechanical properties of 2707 HDSS.

Material	Tensile strength (MPa)	Yield strength 0.2% (MPa)	Elongation (%)
2707 HDSS	950	650	25

**Table 3:** The electrochemical corrosion parameters determined from the polarization curves of 2707 HDSS after 14 days of incubation at 37 ^o^C.

Medium	i_corr_ (μA cm^−2^)	E_corr_ (mV)	*β*_*a*_ (mV/dec)	*β*_*c*_ (mV/dec)
Sterile	0.087	−472	286.4	−237.8
*P. aeruginosa* inoculated	0.328	−277	121.2	−153.6

**Table 4:** Electrochemical model impedance parameters of 2707 HDSS in the *P. aeruginosa* broth and in the abiotic medium.

Duration(days)	R_s_(Ω cm^2^)	Q_CPE_×10^−5^(Ω^−1^ S^n^ cm^−2^)	n	R_b_(kΩ cm^2^)	C_dl_(μF cm^−2^)	R_ct_(kΩ cm^2^)
2707 HDSS in the *P. aeruginosa* broth						
1	9.46	6.416	0.7961	16.48	168.4	24.9
4	8.56	7.791	0.7702	21.65	274.6	29.2
7	9.49	6.476	0.7539	21.68	185.9	29.1
14	7.93	6.446	0.7016	24.61	103.1	32.1
2707 HDSS in the abiotic medium						
1	11.89	4.360	0.8676	—	—	807.2
4	11.01	3.224	0.8405	—	—	388.0
7	11.75	2.979	0.8273	—	—	568.3
14	9.56	2.720	0.7803	—	—	489.4

**Table 5:** Pit depth and biofilm thickness of 2707 HDSS in the abiotic medium and *P. aeruginosa* broth.

	abioticmedium	*P. aeruginosa*incubated for 7 days	*P. aeruginosa*incubated for 14 days
Maximum pit depth (μm)	0.02	0.52	0.69
Average largest pit depth (μm)	0.017 ± 0.01	0.42 ± 0.12	0.52 ± 0.15
Largest biofilm thickness (μm)	—	23.4	18.9
Average biofilm thickness (μm)	—	22.2 ± 0.7	17.8 ± 1.0

**Table 6:** Relative atomic percent (%) of the main constituents on the surface of 2707 HDSS with and without exposure to *P. aeruginosa*: (A) in the abiotic medium for 7 days, (B) in the abiotic medium for 14 days, (C) in the inoculated medium for 7 days, and (D) in the inoculated medium for 14 days.

Sample	Atomic percent (%)							
	Cl	C	N	O	Cr	Fe	Na	Mg
A	4.59	38.81	5.45	36.67	3.69	1.82	7.78	0.22
B	7.39	42.46	5.30	29.64	2.95	1.30	9.65	0.75
C	6.39	58.93	5.76	23.70	0.51	0.39	3.56	0.43
D	2.62	56.95	5.35	28.71	0.21	0.16	5.64	0.36
